# Disabling Dactylitis and Tenosynovitis Due to *Mycobacterium haemophilum* in a Patient With Human Immunodeficiency Virus/Acquired Immune Deficiency Syndrome

**DOI:** 10.1093/ofid/ofx165

**Published:** 2017-09-23

**Authors:** Michael H Woodworth, Carina Marquez, Henry Chambers, Anne Luetkemeyer

**Affiliations:** 1 Infectious Diseases Fellow, Division of Infectious Diseases, Department of Medicine, Emory University School of Medicine, Atlanta, Georgia; 2 Zuckerberg San Francisco General, University of California

**Keywords:** AIDS, HIV, IRIS, *Mycobacterium haemophilum*, nontuberculous mycobacteria

**Figure 1.**
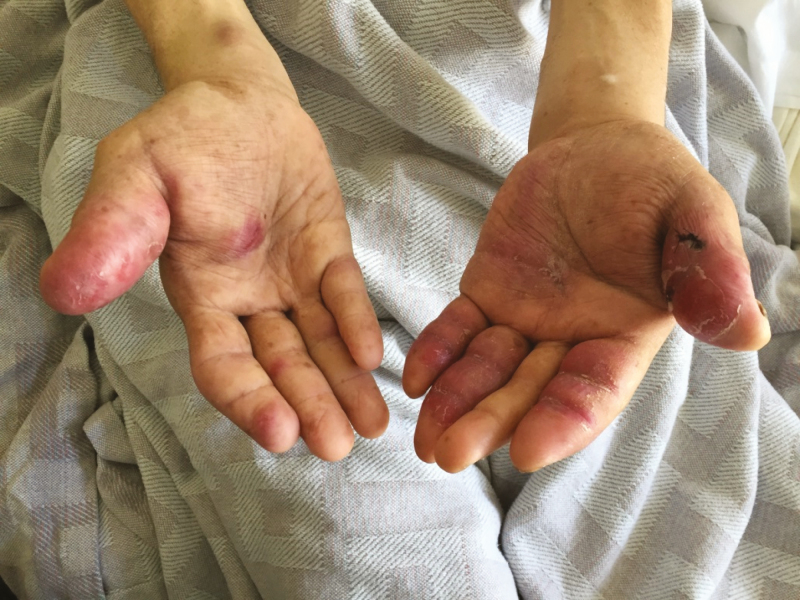
Photograph of patient with acquired immune deficiency syndrome with bilateral, disabling dactylitis and tenosynovitis 1 month after reinitiation of antiretroviral therapy.

**Figure 2.**
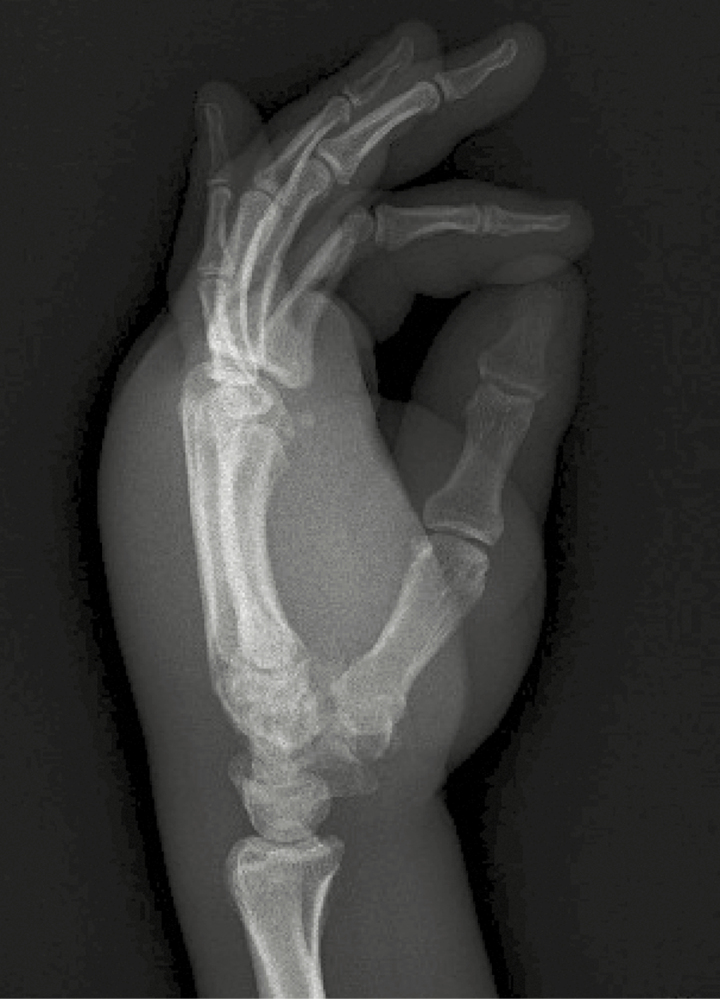
Left hand radiograph without fracture or joint disease illustrating degree of diffuse soft tissue swelling.

**Figure 3.**
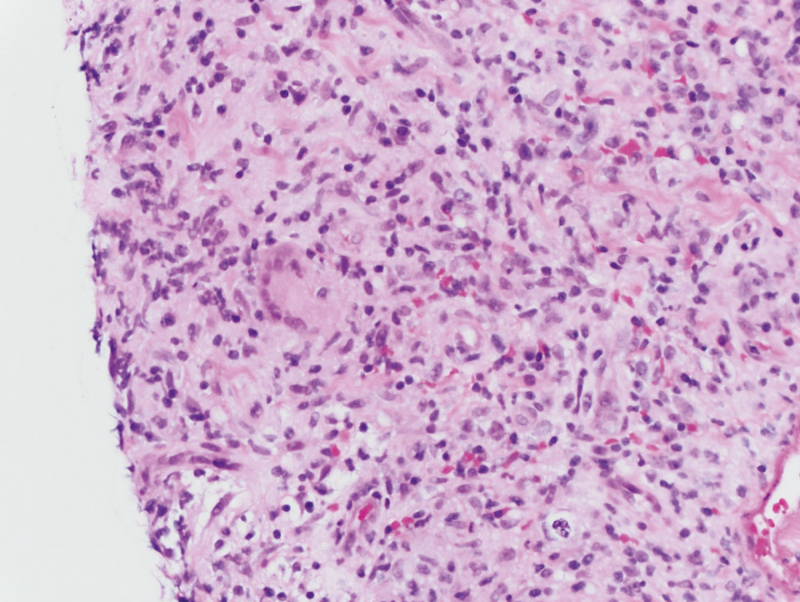
Photomicrograph of left lateral thumb punch biopsy with granulomatous inflammation at the base of the biopsy.

**Figure 4.**
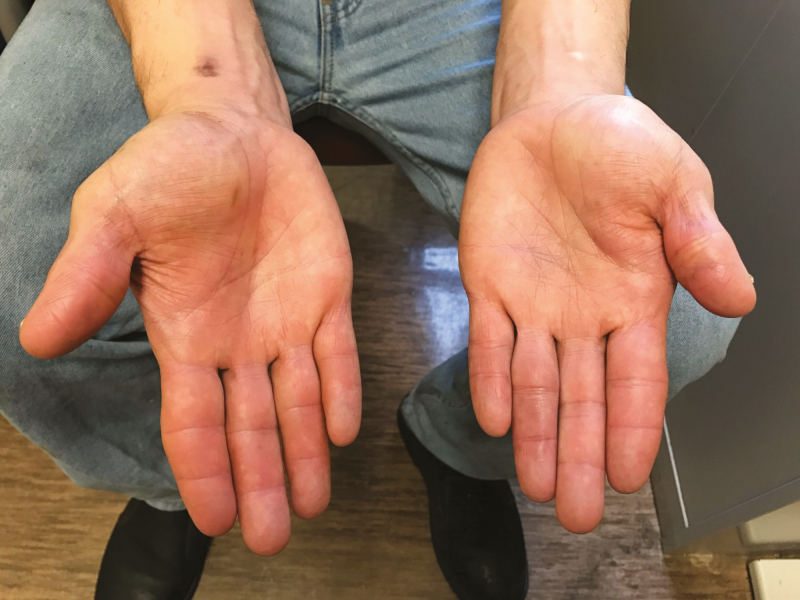
Photograph of patient with acquired immune deficiency syndrome with bilateral, disabling dactylitis and tenosynovitis after 9 months of therapy for *Mycobacterium haemophilum*.

A 44-year-old man with acquired immune deficiency syndrome (AIDS) and CD4^+^ cell count of 8 cells/mm^3^ presented with painful and erythematous swollen hands 1 month after starting antiretroviral therapy (ART) after a 4-year hiatus from care. During his interrupted human immunodeficiency virus (HIV) treatment, he was living in Mexico and incarcerated for a period of time. For the year preceding presentation with hand swelling, he was working as a cook in a Mexican restaurant in San Francisco, California, where he had frequent finger trauma from opening metal cans and preparing shrimp. Thickened flexor tendons and dactylitis were present bilaterally with stiffness and severely limited flexion and extension of his fingers (Figures 1 and 2). He did not have systemic symptoms. Skin biopsies demonstrated granulomatous dermatitis and panniculitis (Figure 3), with negative stains for organisms. Polymerase chain reaction and immunohistochemical stains for mycobacteria were negative. TB treatment along with clarithromycin was initiated empirically. Cultures from a second excisional biopsy of deep palm soft tissue on chocolate agar incubated at 30°C grew *Mycobacterium haemophilum*, suggesting unmasking immune reconstitution inflammatory syndrome (IRIS) in setting of HIV treatment. Additional biopsies grew *M. haemophilium* from mycobacterial cultures. Treatment was changed to moxifloxacin, ethambutol, rifabutin, and azithromycin. Eight weeks later, after initial improvement, hand swelling and pain increased, accompanied by spontaneous drainage of cold abscesses on wrist, elbow, and knee, without evidence of an alternative etiology. Prednisone was administered for paradoxical IRIS for 3 months, with resolution of symptoms. After 9 months of treatment for *M haemophilum*, swelling and tenosynovitis fully resolved to baseline function (Figure 4). Antibiotics were discontinued after 12 months without relapse.

*Mycobacterium haemophilum* is a slow-growing, fastidious organism that grows best at 30°C (like *Mycobacterium marinum* and *Mycobacterium ulcerans*) and requires supplementation of iron or hemin [1]. *Mycobacterium haemophilum* resides in the environment and has been isolated from biofilms in fish tanks and water systems [2]. Infection has also been reported after superficial trauma from coral, which may have been relevant in this patient who handled seafood [2]. Most cases of *M haemophilum* have been reported in immunocompromised patients, including patients with hematologic malignancy, hematopoietic and solid organ transplantation, and HIV/AIDS. Extremity infections are the most common, namely skin and soft tissue infections, tenosynovitis, and osteomyelitis, likely related to improved growth of *M haemophilum* at lower temperatures [1, 3]. *Mycobacterium haemophilum* has been relatively commonly isolated among pediatric mycobacterial lymphadenitis cases when appropriate culture methods are used [4]. Disseminated infection represents severe disease and is usually seen in immunocompromised patients. Paradoxical IRIS in HIV patients with nontuberculous mycobacterial (NTM) infection is not as well described as for *Mycobacterium tuberculosis* (TB). However, clinical similarities between NTM and TB-IRIS have been previously noted, and, in one series, the incidence of NTM-IRIS was 3.5% among patients initiating ART with a baseline CD4 count <100 cells/μL [5]. There are no standardized methods for *M haemophilum* susceptibility testing or interpretation, although there are CLSI recommendations for disk-diffusion susceptibility testing [3]. Surgery may be indicated and dependent on severity of disease and ability to reduce immunosuppression. Prolonged treatment of *M haemophilum* is recommended with 6–24 months of a multidrug regimen that includes a macrolide, fluoroquinolone, and a rifamycin [3].
